# Verbal fluency after cochlear implantation: a longitudinal comparison with untreated hearing loss in the ELSA cohort

**DOI:** 10.3389/fnins.2026.1854922

**Published:** 2026-07-15

**Authors:** Christiane Völter, Lisa Bode, Stefan Dazert, Stefan Thomas Kamin

**Affiliations:** 1 Department of Otorhinolaryngology, Head and Neck Surgery, Catholic Hospital Bochum, Ruhr-University, Bochum, Germany; 2 Department of Otorhinolaryngology, Head and Neck Surgery, Martha-Maria Hospital Halle-Dölau, Halle, Germany; 3 Fraunhofer Institute for Integrated Circuits IIS, Fraunhofer Center for Applied Research on Supply Chain Services SCS, Nuremberg, Germany

**Keywords:** cochlear implantation, dementia, ELSA, hearing loss, verbal fluency

## Abstract

**Introduction:**

Hearing loss is associated with accelerated cognitive decline, and auditory rehabilitation via cochlear implantation (CI) may mitigate this trajectory. In the past, the impact of cochlear implantation on different cognitive subdomains has been described. However, verbal fluency (VF), which requires fast semantic retrieval, executive control, and processing speed, and is predictive of dementia risk and overall survival, has been rarely studied and control groups are mostly missing due to ethical reasons. The present study compares long-term VF trajectories in CI recipients and untreated hearing-impaired controls from a large population-based aging study.

**Materials and methods:**

VF was assessed in 74 CI recipients (M = 65.6 years, SD = 9.1) at pre-operative baseline and 1, 2, 4.5, and up to 9 years post-implantation, and in 383 untreated hearing-impaired participants (M = 72.6 years, SD = 10.0) from the English Longitudinal Study of Ageing (ELSA) across a comparable time frame. Scores were z-standardized within each study to enable cross-cohort comparison. Linear mixed-effects models were used to compare VF trajectories, with age, sex, and education as covariates.

**Results:**

VF trajectories differed significantly between groups (Time × Study interaction: *b* = 0.562, *p* < 0.001). The ELSA cohort showed a steady linear decline over time (*b* = −0.261, *p* = 0.001), whereas the CI cohort exhibited an inverted-U trajectory with initial improvement followed by a plateau. After propensity score matching, results remained robust.

**Conclusion:**

Cochlear implantation is associated with more favorable long-term verbal fluency trajectories compared to untreated hearing loss. These findings add to the growing evidence that auditory rehabilitation may help preserve cognitive function in older adults.

## Introduction

Hearing loss is one of the most prevalent chronic conditions in older adults and has been identified as the largest potentially modifiable risk factor for dementia (Livingston et al., 2024). The mechanism linking hearing loss to cognitive decline remains debated (Merten et al., 2026; Lin, 2020). Leading hypotheses include cognitive load and effortful listening, social isolation and reduced stimulation, and shared neuropathological pathways (common cause) (Powell et al., 2021; Uchida et al., 2019).

Cochlear implants are increasingly provided to older adults when conventional hearing aids no longer provide sufficient benefit (Boisvert et al., 2020). In addition to increased speech understanding observed even in older adults, cochlear implant users also experience significant improvements in psychological well-being, health-related quality of life and social participation (Cuda et al., 2024; Tang et al., 2024; Boisvert et al., 2020; Johnson et al., 2025; Shen et al., 2023; Wang et al., 2024). Recently, growing attention has been directed towards the positive effects of auditory rehabilitation through hearing aids or cochlear implants on various cognitive subdomains, mostly pronounced in attention, inhibition, and working memory (Völter et al., 2022; Andries et al., 2023; Calvino et al., 2022; Huber et al., 2021; Knopke et al., 2021; Mertens et al., 2021; Mosnier et al., 2024; Ohta et al., 2022; Takkoush et al., 2026; Vandenbroeke et al., 2025; Sarant et al., 2024).

However, evidence on cognitive outcomes post-implantation in the long term remains limited (Vandenbroeke et al., 2025; Mosnier et al., 2018; Völter et al., 2023; Takkoush et al., 2026; Amini et al., 2023) and methodologically heterogeneous, with most studies relying on rather small or uncontrolled pre-post designs (Cosetti et al., 2016; Gurgel et al., 2022; Issing et al., 2021; Sarant et al., 2019). One major limitation of the existing literature is the lack of appropriate control groups, largely due to ethical constraints that preclude withholding treatment (Amini et al., 2023; Dawes and Völter, 2023; Völter et al., 2023). Comparing cochlear implant (CI) recipients to individuals with untreated hearing loss in population-based data can help disentangle the natural trajectory of cognitive aging with hearing loss from potential CI-related effects.

Verbal fluency (VF) is a particularly relevant outcome measure: it taps into executive function, semantic memory retrieval, and processing speed, all sensitive to age-related decline while being less dependent on peripheral hearing than other neuropsychological tests (Amunts et al., 2021; Dorchies et al., 2024). Deterioration in semantic fluency is recognized as a risk factor for dementia (Gustavson et al., 2020). Recently, longitudinal data from the Berlin Aging Study (*N* = 516, M = 84.9 years) demonstrated that participants in the upper quartile of verbal fluency performance lived almost 9 years longer than those with lower scores (Ghisletta et al., 2025). VF is routinely collected in large population-based aging studies, such as the English Longitudinal Study of Ageing (ELSA), the Survey of Health, Ageing and Retirement in Europe (SHARE), and the U. S. Health and Retirement Study (HRS), with harmonized longitudinal ageing surveys that allow direct comparison between clinical CI cohorts and population norms (Gkotzamanis et al., 2023; Rikos et al., 2024; Nichols et al., 2026).

This study uses longitudinal data from a consecutive clinical CI cohort (*n* = 74) and participants with untreated hearing loss from the English Longitudinal Study of Ageing (ELSA; *n* = 383) to compare VF trajectories over time.

## Study design and participants

Two independent cohorts of older adults with hearing loss were studied: (a) cochlear implant (CI) recipients who underwent surgical intervention and (b) participants of the English Longitudinal Study of Ageing (ELSA) with objectively measured, untreated hearing loss (i.e., no hearing aid and no cochlear implant).

### CI cohort

The CI sample comprised 74 adults with postlingual bilateral severe-to-profound sensorineural hearing loss and a mean duration of pre-operative deafness of 22.67 (SD 13.42) years who received a cochlear implant at the Ear, Nose, and Throat Department of the Ruhr-University Bochum, Germany. Cognitive assessments were conducted at five time points: pre-implantation (T1) and at approximately 12 (T2), 24 (T3), 54 (T4), and 99 months (T5) post-implantation, spanning a mean follow-up of 8.25 years. Exclusion criteria were insufficient German language proficiency to complete neuropsychological testing and severe neurologic or psychologic impairments. Preoperative speech perception scores in quiet were 6.67% (SD 12.28) correct monosyllabic words at 65dB and 57.64% (SD 21.83) 1 year after implantation as well as 60.55% (SD 20.86) 2 years, 54.39% (SD 20.04) 4.5 years and 62.32% (SD 21.23) 8.25 years post CI.

### ELSA cohort

The comparison sample was drawn from ELSA, an observational nationally representative panel study of community-dwelling adults aged 50 and older in England (Steptoe et al., 2013) including objective hearing measures. We selected participants from five consecutive waves: Wave 7 (fieldwork 2014–2015), Wave 8 (2016–2017), Wave 9 (2018–2019), Wave 10 (2021–2023, with fieldwork partly delayed by the COVID-19 pandemic), and Wave 11 (2023–2024) who met the following inclusion criteria at baseline: (a) objectively measured moderate-to-severe hearing loss, defined as hearing 0–2 of 6 tones on the HearCheck screener in the worse ear; (b) no current hearing aid use; and (c) no cochlear implant. Hearing loss was assessed using the Siemens HearCheck Screener, a handheld device that produces a fixed series of three mid-frequency tones (1 kHz) and three high-frequency tones (3 kHz) at decreasing intensities (Davis et al., 2007). Following the classification used by Ray et al. (2018), hearing acuity was determined based on the worse ear: good (6 tones), mild difficulty (3–5 tones), and moderate-to-severe difficulty (0–2 tones). After applying all inclusion criteria, the ELSA analytic sample comprised 383 adults (206 female [53.8%]; M = 72.6 years, SD = 10.0, range: 50–89). ELSA assessments were nominally biennial; however, the COVID-19 pandemic disrupted the planned fieldwork schedule for Waves 10 and 11 (Lloyd et al., 2023). Because the publicly released ELSA data top-code age at 90 to protect confidentiality, exact ages are unavailable for participants aged 90 or older; we therefore restricted the ELSA sample to participants aged 50–89 at Wave 7.

### Measures

Verbal fluency was assessed in the ELSA cohort using a semantic fluency paradigm in which participants named as many animals as possible within 60 s (Llewellyn and Matthews, 2009). The score was the total number of valid animals named (baseline *M* = 17.7, *SD* = 7.5). In the CI cohort, verbal fluency was assessed in a combined letter and category fluency task paradigm adapted from the Chicago Word Fluency Test (Thurstone, 1948) which was developed and validated for use in hearing-impaired older adults (Völter et al., 2017; Götze et al., 2024), where as many animals as possible starting with a particular letter must be named within 90 s (Völter et al., 2017). The category constraint (animals) engages temporal-semantic retrieval, while the letter constraint adds an executive-frontal filter, so that performance reflects the joint contribution of semantic search and executive selection. Performance was quantified as an inverse efficiency (IE) score, defined as the ratio of processing speed to accuracy (lower IE scores indicate better efficiency). For analysis, IE scores were inverted using vf = (max (IE) + 100) − IE so that higher values indicate better performance. Two re-analyses on raw word counts (on the 90-s window and on the first 60 s of each protocol to match the ELSA response window) were conducted. Baseline word-count statistics for both scoring windows are provided in Supplementary Table S1.

Possible practice effects were minimized by follow-up intervals for cognitive testing spanning at least 1 year and different test versions (Basso et al., 1999).

### Covariates

Because the two cohorts were drawn from different populations and differed in baseline demographic characteristics, three covariates were included in all models: age (centered at 70 years), sex (0 = male, 1 = female), and education level. Education was operationalized as a binary variable separating upper secondary education or higher from lower levels. In the CI cohort, participants with Abitur or higher were classified as high education (*n* = 11, 14.9%); in the ELSA cohort, participants with A-level or university degree were classified as high education (*n* = 109, 28.5%).

### Outcome standardization

Because the two cohorts used different scoring metrics for verbal fluency, direct score comparisons were not possible. To enable cross-study comparison, scores were z-standardized within each study relative to baseline: for each participant, the raw score at each time point was centered on the respective study’s baseline mean and scaled by the baseline standard deviation. This yields scores in baseline SD units (*z* = 0 at the study-specific baseline mean), allowing trajectory comparisons in terms of relative change.

### Statistical analysis

All analyses were conducted in R (R Core Team, 2025) using the lme4 and lmerTest packages (Bates et al., 2015; Kuznetsova et al., 2017).

### Time standardization

To compare trajectories across studies with different follow-up durations, time was rescaled to a 0–1 range, where 0 represents the first assessment and 1 represents the last. A slope of, for example, −0.50 would thus indicate a decline of half a baseline standard deviation from the first to the last assessment.

### Linear mixed-effects models

Two types of growth models were fitted using restricted maximum likelihood (REML): (a) a linear model estimating a constant rate of change, and (b) a quadratic model adding a time^2^ term to capture curvilinear trajectories. All models included random intercepts and random slopes for time at the participant level. Cross-study models included a study indicator (0 = ELSA, 1 = CI) and its interaction with time terms to test whether trajectories differed between groups.

### Sensitivity analysis

To assess the robustness of the primary findings under demographic matching, a propensity score full matching analysis was conducted.

## Results

### Sample characteristics

Table 1 presents the baseline demographic characteristics of both cohorts. The CI cohort was younger (*M* = 65.6 years vs. 72.6 years), included a higher proportion of women (59.5% vs. 53.8%), and had a lower proportion of participants with upper secondary education or higher (14.9% vs. 28.5%). These between-group differences were statistically controlled for in all models.

**Table 1:** Baseline demographic and study characteristics.

Variable	CI cohort	ELSA cohort	Test statistic	*p*
	(*n* = 74)	(*n* = 383)		
Age (years), M (SD)	65.6 (9.1)	72.6 (10.0)	*t* = −5.97	<0.001
Range	50–84	50–89		
Sex (female), *n* (%)	44 (59.5)	206 (53.8)	χ^2^ (1) = 0.59	0.441
Education (high)^a^, *n* (%)	11 (14.9)	109 (28.5)	χ^2^ (1) = 5.24	0.022
Verbal fluency (raw), M (SD)	214.7 (74.5)^b^	17.7 (7.5)^c^	–^d^	–

CI, cochlear implant; ELSA, English Longitudinal Study of Ageing. Group comparisons: Welch’s *t*-test for continuous variables, χ^2^ test (Yates-corrected) for categorical variables.

a CI: educational years >11 (German Abitur); ELSA: qual2 = 1 (A-level or higher).

b Inverse efficiency score (transformed; higher = better performance).

c Number of animals named in 60 s.

d Not compared: different scoring metrics across cohorts (inverse efficiency vs. count).

### Cross-study comparison of verbal fluency trajectories

#### Linear model

The linear mixed-effects model revealed a significant Time × Study interaction (*b* = 0.562, SE = 0.167, *p* < 0.001). The main effect of time indicated a significant decline in the ELSA reference group (*b* = −0.261, SE = 0.080, *p* = 0.001), while the CI cohort’s trajectory was 0.56 SD more favorable over the same standardized timeframe.

#### Quadratic model

The quadratic model revealed significant interactions for both the linear (Time × Study: *b* = 3.636, *SE* = 0.469, *p* < 0.001) and the quadratic (Time^2^ × Study: *b* = −3.483, *SE* = 0.501, *p* < 0.001) components. In the ELSA cohort, the quadratic time term was not significant (*b* = 0.044, *SE* = 0.232, *p* = 0.850). The significant cross-study quadratic interaction reflects the curvilinear trajectory in the CI cohort.

#### Within-study trajectories

**Figure 1:**
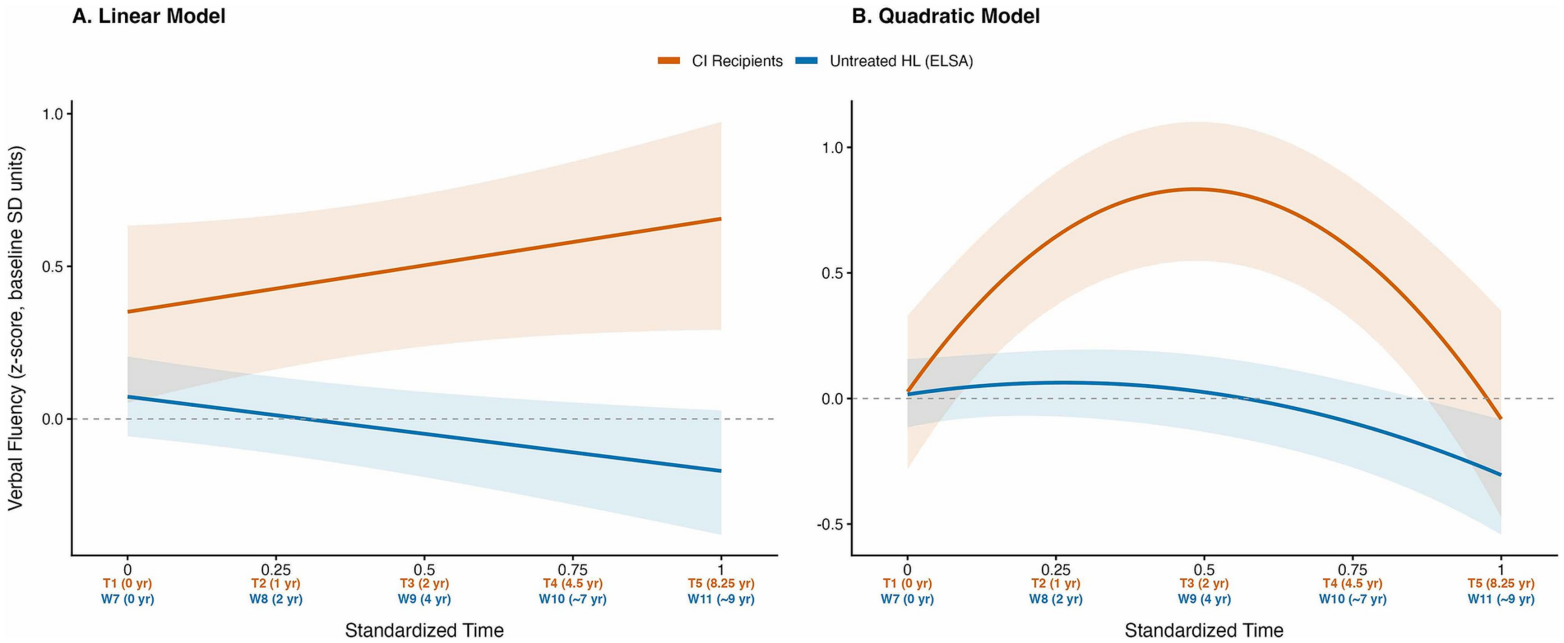
Predicted change in verbal fluency by linear model **(A)** and by quadratic model **(B)** for CI cohort (orange) and for ELSA cohort (blue) controlled for age, sex and education. A higher score represents a better performance. Time was rescaled to a 0–1 range, 0 represents the first assessment and 1 represents the last. For example, a slope of −0.50 indicates a decline of half a baseline standard deviation from baseline to the last assessment.

For the CI cohort, the quadratic model provided a better fit (ΔAIC = 29.2 in favor of the quadratic model). The significant positive linear term (*b* = 3.288, *SE* = 0.557, *p* < 0.001) combined with the significant negative quadratic term (*b* = −3.374, *SE* = 0.591, *p* < 0.001) describes an inverted-U trajectory (Figure 1). For the ELSA cohort, the linear model was preferred (ΔAIC = −3.4, indicating no improvement from the quadratic term; quadratic term: *b* = 0.023, *SE* = 0.201, *p* = 0.911). The linear model revealed a significant decline of *b* = −0.292, *SE* = 0.076, *p* < 0.001, corresponding to approximately 0.29 baseline SD units over the standardized follow-up period. This within-study model selection mirrors the significant cross-cohort interaction.

### Covariate effects

In the cross-study linear model, higher education was associated with better verbal fluency performance (*b* = 0.434, *SE* = 0.096, *p* < 0.001), and older age was associated with lower performance (*b* = −0.025 per year, *SE* = 0.004, *p* < 0.001). Sex was not significantly associated with verbal fluency in the cross-study model (*b* = −0.033, *SE* = 0.084, *p* = 0.693). Within-study analyses revealed opposing sex effects: in the CI cohort, women performed significantly better than men (*b* = 0.844, SE = 0.229, *p* < 0.001), while in the ELSA cohort, women performed significantly worse (*b* = −0.214, SE = 0.087, *p* = 0.014). Full model coefficients are presented in Tables 2, 3.

**Table 2:** Cross-study mixed-effects models for verbal fluency (*N = 457; 1,411 observations*).

Parameter	Linear model			Quadratic model		
	*b*	SE	*p*	*b*	SE	*p*
Intercept	−0.040	0.074	0.584	−0.035	0.076	0.639
Time	−0.261	0.080	0.001	−0.305	0.216	0.158
Time^2^	–	–	–	0.044	0.232	0.850
Study (CI)	0.243	0.123	0.049	−0.086	0.131	0.512
Age (centered)	−0.025	0.004	<0.001	−0.026	0.004	<0.001
Sex (female)	−0.033	0.084	0.693	−0.037	0.084	0.664
Education (high)	0.434	0.096	<0.001	0.434	0.096	<0.001
Time × Study	0.562	0.167	<0.001	3.636	0.469	<0.001
Time^2^ × Study	–	–	–	−3.483	0.501	<0.001

Time = standardized time (0–1). Study coded 0 = ELSA, 1 = CI. Age centered at 70 years. SE, standard error; b, unstandardized fixed-effect estimate; p, p-value from t-tests with Satterthwaite-approximated degrees of freedom. Statistical significance was evaluated at a two-tailed α of 0.05.

**Table 3:** Within-study mixed-effects models for VF (CI cohort; n = 74; 295 observations; ELSA cohort; n = 383; 1,116 observations).

Parameter	CI cohort						ELSA cohort					
	Linear model			Quadratic model			Linear model			Quadratic model		
	*b*	SE	*p*	*b*	SE	*p*	*b*	SE	*p*	*b*	SE	*p*
Intercept	−0.221	0.211	0.296	−0.551	0.218	0.013	0.077	0.073	0.293	0.078	0.074	0.293
Time	0.290	0.198	0.145	3.288	0.557	<0.001	−0.292	0.076	<0.001	−0.311	0.186	0.095
Time^2^	–	–	–	−3.374	0.591	<0.001	–	–	–	0.023	0.201	0.911
Age (centered)	−0.002	0.012	0.881	−0.003	0.013	0.798	−0.030	0.004	<0.001	−0.030	0.004	<0.001
Sex (female)	0.844	0.229	<0.001	0.832	0.231	<0.001	−0.214	0.087	0.014	−0.214	0.087	0.014
Education (high)	0.469	0.311	0.136	0.513	0.314	0.107	0.400	0.096	<0.001	0.400	0.096	<0.001

Time = standardized time (0–1). Age centered at 70 years. SE, standard error; b, unstandardized fixed-effect estimate; p, p-value from t-tests with Satterthwaite-approximated degrees of freedom. Statistical significance was evaluated at a two-tailed α of 0.05.

### Attrition

Both cohorts showed attrition over the follow-up period, with lower retention from Wave 10 onward, coinciding with the COVID-19 pandemic. In the CI cohort, retention was high through 24 months (T3: 72 of 74; 97.3%) but declined to 49 (66.2%) at T4 and 28 (37.8%) at T5. In the ELSA cohort, retention decreased from 281 at Wave 8 (73.4%) to 100 at Wave 11 (26.1%). Mixed-effects models used all available observations (1,411 total across both cohorts), thereby reducing the impact of attrition. To assess potential selective attrition, baseline verbal fluency scores were compared between participants retained at the final assessment and those who dropped out earlier. In the ELSA cohort, retained participants had significantly higher baseline scores than dropouts (M = 21.6, SD = 6.0 vs. M = 16.3, SD = 7.5; t = 7.14, *p* < 0.001, d = 0.75), indicating selective attrition of lower-performing participants. In the CI cohort, no significant difference was found (t = 0.72, *p* = 0.472, d = 0.16). Per-wave sample sizes and word-count descriptives for both scoring windows are reported in Supplementary Table S2 (90-s window) and Supplementary Table S3 (60-s window).

### Sensitivity analysis

#### Real-time axis

Findings remained stable when the cross-cohort quadratic model was re-fitted on a real-time axis (CI: 0, 1, 2, 4.5, 8.25 years; ELSA: 0, 2, 4, 7, 9 years): the linear (time_years × study_ci: *b* = 0.345, *p* < 0.001) and quadratic (time_years^2^ × study_ci: *b* = −0.043, *p* < 0.001) interaction terms remained significant (Supplementary Table S4).

#### Propensity score matching

To assess whether the primary findings were robust to direct demographic matching, a propensity score full matching analysis was conducted using the MatchIt package (Ho et al., 2011). Propensity scores were estimand via logistic regression with age, sex, and education (binary) as covariates, using the average treatment effect on the treated (ATT) as the estimand. Full matching yielded 73 subclasses retaining all 74 CI and 383 ELSA participants; all post-matching standardized mean differences were below 0.10 (largest: age, SMD = 0.072). The effective sample size (ESS) on the ELSA side was 131.6. Results were consistent with the primary analysis: the linear Time × Study interaction was *b* = 0.557, *SE* = 0.191, *p* = 0.004, and the quadratic interactions remained highly significant (Time × Study: *b* = 3.438, *SE* = 0.459, *p* < 0.001; Time^2^ × Study: *b* = −3.344, *SE* = 0.489, *p* < 0.001). The direction and significance of all effects were consistent across both approaches, supporting the robustness of the findings.

## Discussion

Our study compared verbal fluency trajectories in CI recipients with a control group of individuals with untreated hearing loss over a follow-up period of up to 9 years. The central finding is that VF trajectories differed significantly between the two groups: whereas the ELSA cohort with untreated hearing loss showed a steady linear decline, the CI cohort exhibited an inverted-U trajectory, with initial improvement followed by a gradual return toward baseline levels. Over the full standardized follow-up, the CI cohort’s trajectory was 0.56 SD more favorable than that of the untreated hearing loss group. This is important as VF is significantly linked to longevity, even if the explanation is not clear (Ghisletta et al., 2025).

Verbal fluency (VF) is a particularly complex cognitive ability that requires an interplay of different cognitive abilities (Schmidt et al., 2017). Fluency tasks have been shown to be especially sensitive to prefrontal and frontal-subcortical deficits, to mild cognitive impairment and to dementia diagnosis (García-Herranz et al., 2020). The processing strategies for semantic and phonemic fluency tasks are different, whereas semantic fluency correlates with temporal, phonemic fluency is linked to frontal lobe regions and a decline of VF in aging seems to be more pronounced in semantic than in phonemic fluency (Gordon et al., 2018).

The present study is, to our knowledge, the first to compare VF trajectories in CI recipients with a control group of individuals with untreated hearing loss over a follow-up of up to 9 years. It adds further data to the growing body of literature suggesting a positive impact of CI on the cognitive functioning of individuals with hearing loss.

So far, VF has been rarely studied in CI cohorts and with mixed results (Baranger et al., 2023; Huber et al., 2025; Mosnier et al., 2018; Cosetti et al., 2016; Völter et al., 2022). Huber et al. reported a significant improvement in semantic fluency 12 months after implantation in 21 older CI recipients (aged 60–75 years), with an average gain of 3.71 words (range: 1.65–5.78), but no improvement was found in letter fluency or in a younger subgroup (*n* = 20, aged 25–58 years) (Huber et al., 2025). In contrast, Baranger et al. reported an improvement only in phonemic fluency 3 months after CI in a mixed cohort of 43 individuals with congenital and acquired hearing loss (Baranger et al., 2023). The present study extends these findings by providing long-term data beyond the typical 12-month follow-up and by including an external comparison group, addressing a key methodological limitation of prior work (Takkoush et al., 2026; Amini et al., 2023; Dawes, 2019; Völter et al., 2022).

A plausible pathway linking CI to improved VF is by an increase in working memory through diminished cognitive load as described in detail by the Ease of Language Understanding Model, published by Rönnberg et al. (2013). In the past, improvements in different cognitive subdomains after CI have been observed by various research groups, mostly in attentional processes and working memory (Völter et al., 2022; Andries et al., 2023; Calvino et al., 2022; Huber et al., 2021; Knopke et al., 2021; Mertens et al., 2021; Mosnier et al., 2024; Ohta et al., 2022; Takkoush et al., 2026; Vandenbroeke et al., 2025; Sarant et al., 2024). However, Huber et al., who recently studied verbal fluency in younger and older hearing impaired 12 months after cochlear implantation showed that improvement in semantic fluency correlated with enhanced working memory after CI, but that working memory did not fully mediate the effect of CI on semantic fluency (Huber et al., 2025).

A complementary mechanism is through increased social participation (Issing et al., 2024), given that effective communication is central to social activities (Rehan and Phillips, 2025) and that reduced social engagement is itself a dementia risk factor (Sommerlad et al., 2023).

Because social participation was not measured in either cohort, this mechanism remains a hypothesis to be tested in future studies with direct social-engagement assessment (Rehan and Phillips, 2025; Pichora-Fuller et al., 2015; Sommerlad et al., 2023).

The opposing sex effects across cohorts call for caution. Possible explanations include differential referral patterns into the CI cohort, cross-national differences in verbal-fluency performance, and task-format effects (letter-constrained vs. unconstrained fluency). The present data cannot distinguish among these accounts; we regard this finding as exploratory.

### Limitations

This study has four limitations. First, the two cohorts used different verbal fluency tasks (CI: letter-constrained category fluency, 90 s; ELSA: unconstrained semantic fluency, 60 s) and different hearing assessments (audiometric testing vs. HearCheck screener; Davis et al., 2007). Cross-cohort inference therefore rests on the shape of the longitudinal trajectories rather than on absolute score levels. Letter and category fluency share executive-frontal and semantic-temporal substrates and correlate in older adults (Shao et al., 2014; Henry and Crawford, 2004), and a 60-s reanalysis of the CI protocol reproduces the cross-cohort divergence in trajectory shape.

Second, residual confounding cannot be excluded. While age, sex, and education were measured comparably and controlled for in all models, other variables linked to cognitive trajectories (e.g., comorbidity burden, depressive symptoms, physical activity, social engagement) could not be harmonized across cohorts. Within-cohort z-standardization likewise cannot fully eliminate cross-national differences between the German CI cohort and the English ELSA cohort (educational systems, healthcare access, language-specific verbal fluency norms). We therefore read our findings as describing an observed association rather than a controlled CI effect estimate.

Third, both cohorts showed attrition. In the CI cohort, the SD of word counts narrowed from 3.18 at T2 to 1.70 at T5, indicating selective retention of higher-performing participants. Such attrition can attenuate late-wave estimates but cannot generate the early rise from T1 to T3, where attrition was minimal.

Finally, because this study is observational, we cannot draw causal inferences; factors associated with the decision to seek implantation may have shaped the CI cohort’s trajectory.

### To summarize

Considering that hearing loss is associated with a faster cognitive decline, the observation that VF improves after implantation is promising. As it is unethical to deny a cochlear implantation to a severely hearing-impaired person for many years, a fictive control group such as the ELSA cohort might be a good option adding another view to the ongoing discussion on the impact of cochlear implantation on cognition in the long-term. However, there are limitations, and the present data cannot resolve the question whether the cognitive booster effect after implantation might help to prevent or at least to delay dementia. Multicenter studies with larger sample sizes and a long-term follow-up are mandatory to underline this important finding in the context of healthy aging.

## Data Availability

The original contributions presented in the study are included in the article/Supplementary material, further inquiries can be directed to the corresponding author.
