# Supplementary material for: Verbal fluency after cochlear implantation: a longitudinal comparison with untreated hearing loss in the ELSA cohort
Source: Front Neurosci. 2026 Jul 15;20:1854922. doi: 10.3389/fnins.2026.1854922 (PMC13415579; doi:10.3389/fnins.2026.1854922)
Supplement: Supplementary file 1 [file Data_Sheet_1.docx]

**Supplementary**

**Table S1. Baseline verbal-fluency word-count statistics, both scoring windows**

| **Cohort** | **Window** | **Baseline n** | **Mean (words)** | **SD (words)** |
| --- | --- | --- | --- | --- |
| Cochlear (T1) | 90 s | 74 | 6.58 | 2.50 |
| Cochlear (T1) | 60 s | 74 | 4.16 | 1.89 |
| ELSA (W7) | 60 s | 383 | 17.69 | 7.51 |

**Table S2. Per-wave sample sizes and word-count descriptives (90-second window).**

| Cohort | Wave | n observed | Mean (words) | SD (words) |
| --- | --- | --- | --- | --- |
| Cochlear | T1 (0 mo) | 74 | 6.58 | 2.50 |
| Cochlear | T2 (12 mo) | 72 | 8.00 | 3.18 |
| Cochlear | T3 (24 mo) | 72 | 8.75 | 2.99 |
| Cochlear | T4 (54 mo) | 49 | 7.43 | 2.75 |
| Cochlear | T5 (99 mo) | 28 | 7.32 | 1.70 |
| ELSA | W7 (2014/15) | 383 | 17.69 | 7.51 |
| ELSA | W8 (2016/17) | 281 | 17.78 | 7.66 |
| ELSA | W9 (2018/19) | 230 | 17.93 | 7.58 |
| ELSA | W10 (2021/23) | 122 | 18.46 | 8.73 |
| ELSA | W11 (2023/24) | 100 | 20.11 | 8.19 |

*Note. The cross-wave increase in observed ELSA means reflects selective attrition rather than within-person improvement: participants retained at W11 had higher baseline scores than dropouts. Mixed-effect models account for between-person baseline differences via random intercepts and estimate the negative within-person slope reported in the Results (Tables 2 and 3).* A *reanalysis using raw word counts as the outcome reproduced the primary findings: the cross-cohort Time × Study interaction was significant in both the linear (b = 0.60, p < .001) and quadratic (Time² × Study: b = −2.96, p < .001) specifications, and the quadratic model improved fit over the linear model (likelihood-ratio test: χ²(2) = 49.01, p < .001; ΔAIC = 45.0).*

**Table S3.** Per-wave sample sizes and word-count descriptives (60-second window, ELSA-harmonised).

| **Cohort** | **Wave** | **n observed** | **Mean (words)** | **SD (words)** |
| --- | --- | --- | --- | --- |
| Cochlear | T1 (0 mo) | 74 | 4.16 | 1.89 |
| Cochlear | T2 (12 mo) | 71 | 5.18 | 2.19 |
| Cochlear | T3 (24 mo) | 72 | 5.72 | 1.90 |
| Cochlear | T4 (54 mo) | 48 | 4.75 | 1.93 |
| Cochlear | T5 (99 mo) | 28 | 4.93 | 1.44 |
| ELSA | W7 (2014/15) | 383 | 17.69 | 7.51 |
| ELSA | W8 (2016/17) | 281 | 17.78 | 7.66 |
| ELSA | W9 (2018/19) | 230 | 17.93 | 7.58 |
| ELSA | W10 (2021/23) | 122 | 18.46 | 8.73 |
| ELSA | W11 (2023/24) | 100 | 20.11 | 8.19 |

*Note. The cross-wave increase in observed ELSA means reflects selective attrition rather than within-person improvement: participants retained at W11 had higher baseline scores than dropouts. Mixed-effect models account for between-person baseline differences via random intercepts and estimate the negative within-person slope reported in the Results (Tables 2 and 3). A reanalysis using raw word counts (z-standardized within cohort) on the ELSA-harmonised 60-second window reproduced the primary findings: the cross-cohort Time × Study interaction was significant in both the linear (b = 0.61, p < .001) and quadratic (Time² × Study: b = −2.66, p < .001) specifications, and the quadratic model improved fit over the linear model (likelihood-ratio test: χ²(2) = 39.83, p < .001; ΔAIC = 35.8).*

**Table S4. Cross-study mixed-effects model on real-time axis — quadratic specification.**

| Term | b | SE | df | p |
| --- | --- | --- | --- | --- |
| (Intercept) | −0.044 | 0.077 | 549.79 | .564 |
| time_years | −0.040 | 0.025 | 964.07 | .110 |
| time_years² | 0.002 | 0.003 | 1003.95 | .554 |
| study_ci | 0.111 | 0.131 | 519.85 | .397 |
| age_centered | −0.026 | 0.004 | 455.12 | < .001 |
| female | −0.023 | 0.085 | 439.32 | .789 |
| edu_high | 0.443 | 0.097 | 435.78 | < .001 |
| **time_years × study_ci** | **0.345** | 0.063 | 896.21 | **< .001** |
| **time_years² × study_ci** | **−0.043** | 0.008 | 935.32 | **< .001** |

*b, unstandardized fixed-effect estimate; SE, standard error; p, p-value from t-tests with Satterthwaite-approximated degrees of freedom. Statistical significance was evaluated at a two-tailed α of 0.05.*
